# MicroRNAs in circulating extracellular vesicles as biomarkers of early colorectal cancer captured using high mannose *N*-glycan-specific lectin from *Oscillatoria Agardhii*

**DOI:** 10.3389/fonc.2025.1619460

**Published:** 2025-07-30

**Authors:** Sanae Nakayama, Miyabi Umeda, Kenya Kobayashi, Yukiko Nakano, Kanji Hori, Tsukuru Umemura, Hiroshi Kurokawa

**Affiliations:** ^1^ R&D Department, Alps Alpine Co., Ltd, Osaki, Japan; ^2^ Laboratory of Marine Bioresource Chemistry, Graduate School of Integrated Sciences for Life, Hiroshima University, Higashi-Hiroshima, Japan; ^3^ Graduate School, Department of Medical Technology and Sciences, International University of Health and Welfare, Okawa, Japan; ^4^ Clinical Laboratory, Kouhoukai Takagi Hospital, Okawa, Japan

**Keywords:** OAA, lectin, high-mannose type N-linked glycan, extracellular vesicle, miRNA, colorectal cancer, liquid biopsy, biomarker

## Abstract

**Introduction:**

Lectin (OAA), isolated from the filamentous cyanobacterium *Oscillatoria agardhii*, exhibits high specificity and strong binding affinity for high-mannose (HM) *N*-glycans. Previous studies have demonstrated that OAA captured extracellular vesicles (EVs) derived from cancer cell lines. This study aimed to confirm the effectiveness of OAA in capturing HM *N*-glycans in blood and explore its potential in capturing circulating EVs derived from early-stage colorectal cancer (CRC) tumors.

**Methods:**

OAA1 (a recombinant OAA variant) was used to capture HM *N*-glycans from blood samples. The ability of OAA1 to capture circulating EVs in patients with stage I CRC was assessed. The miRNA profiles of the OAA1-captured EVs were analyzed and compared between 60 patients with stage I CRC and 60 healthy controls. Statistical analyses were performed to evaluate the potential of the specific miRNAs as CRC biomarkers.

**Results:**

OAA1 effectively captured HM *N*-glycans in the plasma. Nanoparticle and immunoblot analyses confirmed the presence of EVs in the OAA1-captured from plasma. The miRNA profile of OAA1-captured EVs exhibited characteristics of patients with CRC. Statistical analysis identified five miRNAs (miR-122-5p, miR-130a-3p, miR-146a-5p, miR-15b-5p, and miR-126) and three internal control miRNAs (miR-93-5p, miR-192-5p, and miR-502-5p) with a high potential for cancer separation (area under the curve (AUC) = 0.948; sensitivity = 0.883; specificity = 0.933).

**Discussion:**

These results suggest that circulating miRNAs in OAA1-captured EVs could serve as biomarkers for the surveillance of early stage CRC using liquid biopsy. The OAA1-immobilized column device facilitates easier and quicker inspection processes and accentuates differences in circulating miRNAs associated with the disease.

**Conclusion:**

OAA1-column showed potential clinical application to analyze circulating EVs and miRNAs associated with CRC, serving as a relevant liquid biopsy for early cancer detection.

## 1 Introduction

High-mannose-type Asparagine (*N*)-linked glycan (HM *N*-glycan) is an early glycan structure involved in the biosynthesis of *N*-linked glycoproteins (1). In tumor cells, *N*-glycosylation biosynthesis in the endoplasmic reticulum (ER) may involve less processing, leading to the accumulation of HM *N*-glycans in the ER, as observed in colorectal cancer (CRC) tissues (2). Several studies have investigated the alterations in glycosylation in various cancers, focusing on these changes as potential cancer markers for developing treatment or prevention methods (3). HM *N*-glycans display distinct patterns throughout CRC development, with a particularly high abundance in early CRC tissues (4). We previously isolated a potent anti-human immunodeficiency virus (HIV) lectin, OAA, from the filamentous cyanobacterium *Oscillatoria agardhii* (5, 6). OAA and its recombinant form produced in *Escherichia coli* exhibit high specificity and affinity for HM *N*-glycans (5–7). OAA-immobilized beads capture extracellular vesicles (EVs) in cancer cell line-cultured medium, but not in normal cell lines (1). These findings suggest that tumor-derived EVs may possess HM *N*-glycans on their surfaces.

MicroRNAs (miRNAs) are short non-coding RNAs, 18–25 nucleotides in length, that regulate gene expression post-transcriptionally across a wide range of organisms, from viruses to humans. Since their first report in 1993 (8), miRNAs have garnered significant interest from researchers as key regulators of epigenetic processes as well as potential biomarkers for various diseases. In 2007, Valadi et al. reported that miRNAs in EVs are released from cells and can be delivered to other cells (9). This suggests that miRNAs play a role in cell-to-cell communication via intercellular gene regulation. EVs, which are released from all cell types into body fluids and circulate throughout the body, contain cell-derived biomolecules such as RNAs, proteins, and metabolites. Several studies have shown that EVs retain the surface characteristics of their derived cells, including surface antigens, protein modifications, and glycosylated molecules. Consequently, EVs and their components, particularly miRNAs, are considered promising biomarkers for human diseases (10, 11), including CRC (12–14).

CRC is the third most common cancer in men and the second most common cancer in women worldwide, with over 1.95 million new cases reported in 2020 (15). In Japan, colon/rectum cancer will be the leading cause of cancer deaths among women in 2022, accounting for 16% of deaths (16). The National Cancer Center of Japan reported the 5-year relative survival rates for CRC diagnosed in 2013–2014 across member hospitals of the Association of Clinical Cancer Centers in Japan: stage I, 94.5%; stage II, 88.4%; stage III, 77.3%; stage IV, 18.7%; and overall, 72.5% (17, 18). The sharp decline in the 5-year relative survival at stage IV underscores the importance of early detection and treatment of CRC.

In this study, we aimed to capture circulating CRC-derived EVs using an OAA1-immobilized column. Profiling of miRNAs in OAA1-captured EVs revealed miRNA signature characteristics of patients with CRC. Statistical analyses of the results from 60 patients with stage I CRC and 60 healthy controls identified candidate miRNAs as biomarkers of early stage CRC.

## 2 Materials and methods

### 2.1 Clinical samples

**Figure 1:**
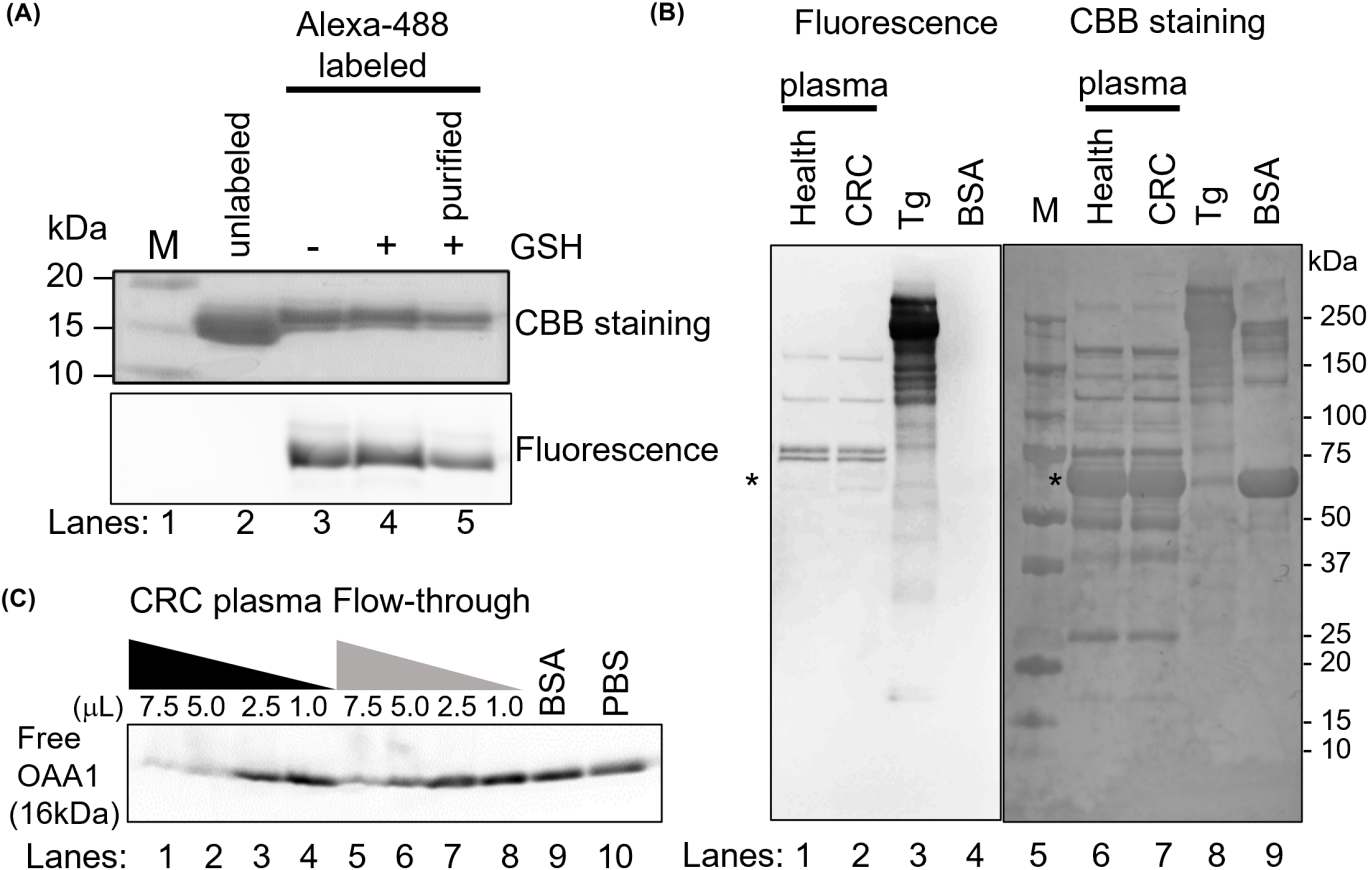
OAA1 bound to HM *N*-glycans in human plasma. **(A)** Labeling of OAA1 with Alexa-488. The labeling reaction was stopped by adding of glutathione (GSH), and the free fluorescent dyes were removed using 3K column (purified). **(B)** Lectin blot analysis using Alexa-488 labeled OAA1 (left) and CBB staining (right) of human plasma (total protein 5 µg each). Purified pig thyroglobulin (Tg) and bovine serum albumin (BSA) were used as positive and negative controls, respectively. The sizes of the molecular weight marker (M) (lane 5) are shown on the right. Asterisks indicate the position of human serum albumin (66.5 kDa). **(C)** Electrophoretic mobility shift assay using fluorescence labeled OAA1. Sixty microliters of CRC plasma were applied to an OAA1-column and the flow-through was collected. Each microliter (7.5, 5.0, 2.5, or 1.0) of CRC plasma or the flow-through, 7.5 µL of BSA or PBS, was mixed with 2.5 µg of the fluorescence-labeled OAA1 and incubated at 37°C for 30 min. Each mixture was separated by SDS-PAGE, and the fluorescence was scanned using the FLA7000. The fluorescence intensity of unbound OAA1 (free OAA1) was measured using ImageJ; the relative values are summarized in **Table 1**.

**Figure 2:**
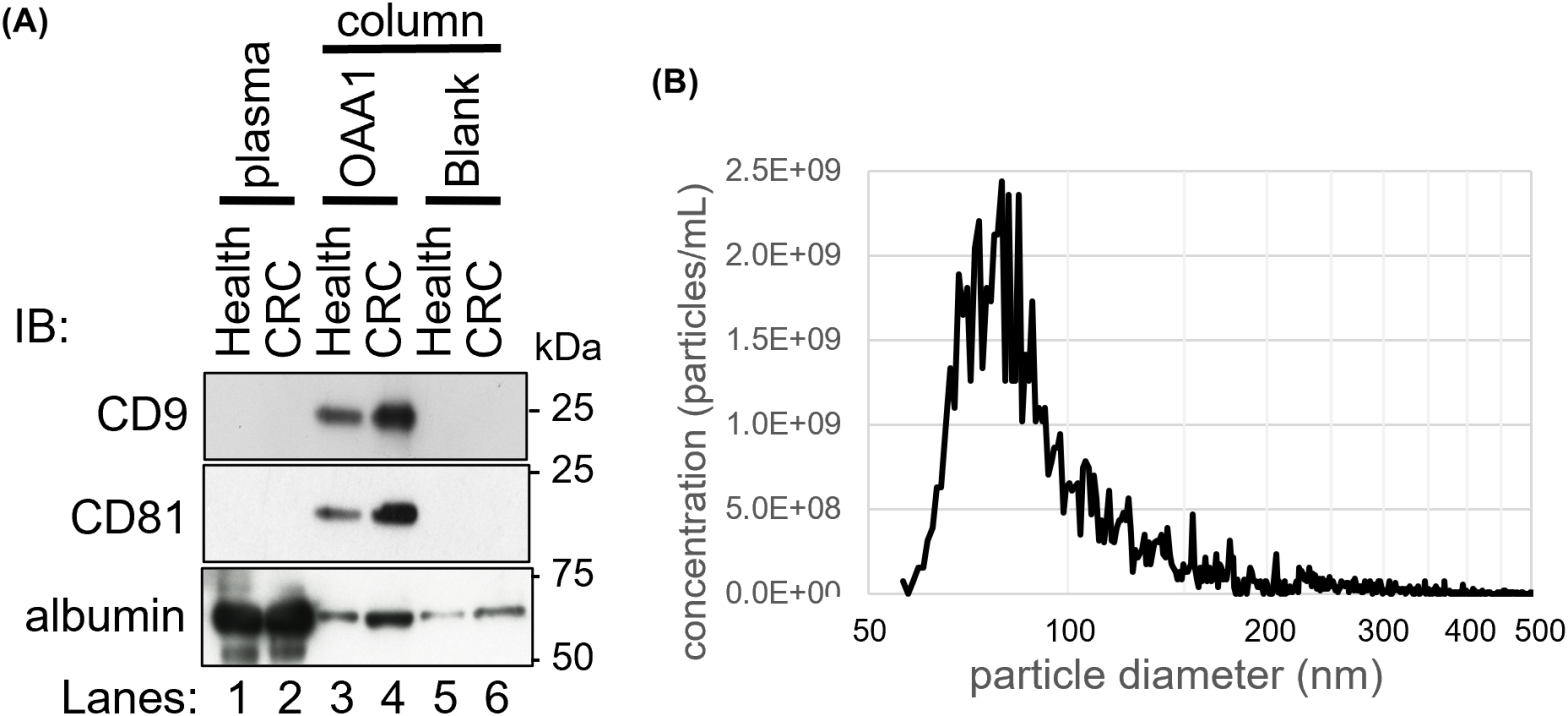
OAA1-captured EVs of human plasma. **(A)** Immunoblot analysis of the proteins captured by the OAA1-column using EV markers (anti-CD9, anti-CD81 monoclonal antibodies), and anti-albumin polyclonal antibodies. Total plasma protein extracts (10 µg for EV markers and 1 µg for albumin), and the eluate from the OAA1-column (20 µL for EV markers, 5 µL for albumin) were separated on a 12.5% SDS-PAGE gel. IB, Immuno bodies. **(B)** Counting the number of particles in OAA1-captures by Tunable Resistive Pulse Sensor (qNano).

**Figure 3:**
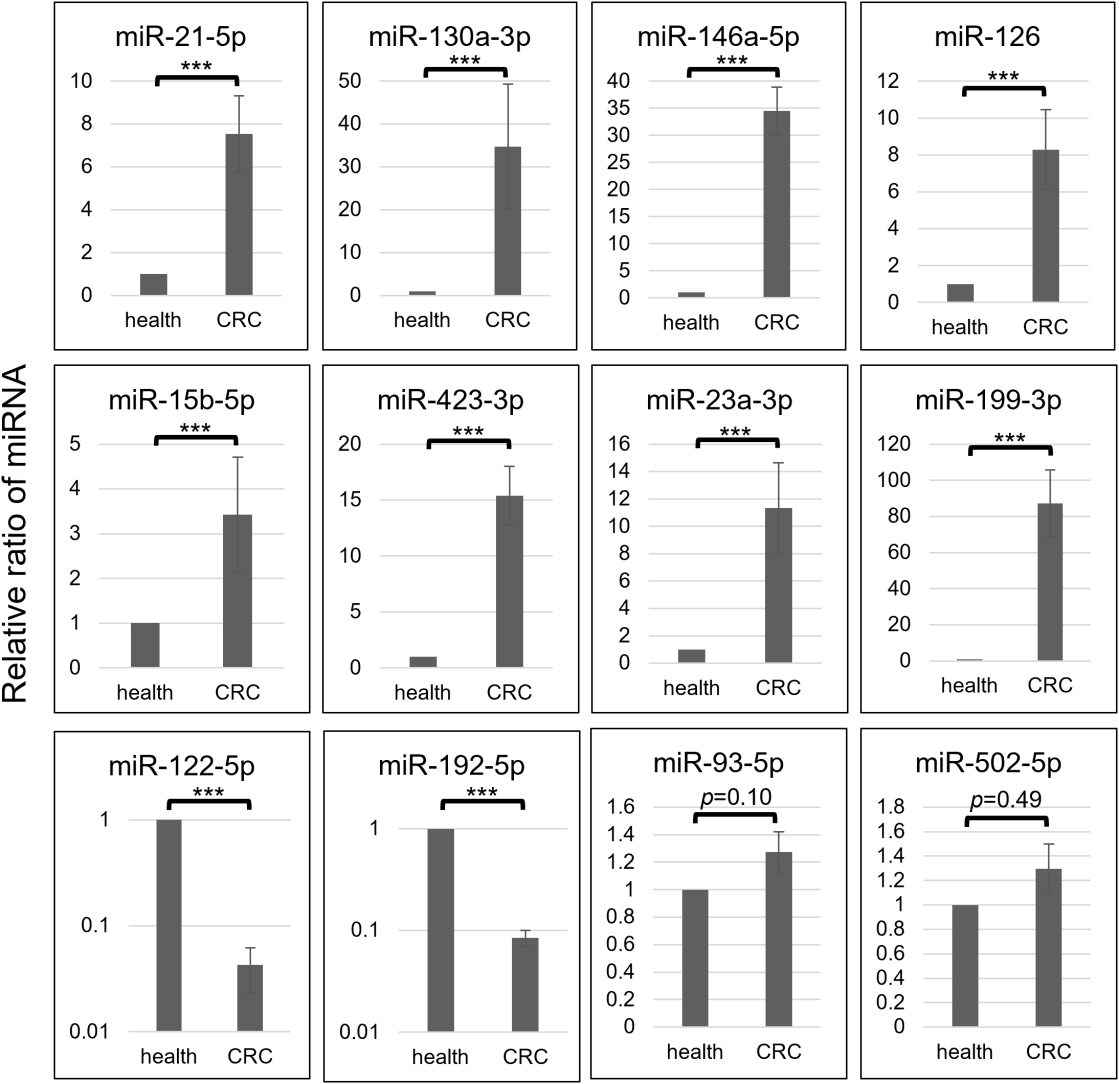
Each selective miRNA abundance in OAA1-captures from pooled healthy control (health) and that from pooled CRC (stage II-IV) plasma (CRC) is shown in relative ratio. Data represent means ± SD of four independent experiments. Statistical significance was determined using an unpaired two-sided Student’s t-test (***p < 0.001).

The CRC staging is based on the postoperative pathological stage, according to guidelines issued by the Japanese Society for Cancer of the Colon and Rectum (19). EDTA-treated plasma from 60 patients with stage I CRC was used for statistical analysis, and 10 patients with CRC (stages II–IV) used for fundamental research (**Figures 1**, **2**, **Table 1**) were purchased from KAC Co., Ltd. (Kyoto, Japan) and International Bioscience Inc. (Tokyo, Japan), respectively. Seventy healthy control samples (EDTA-treated plasma) were obtained with the approval of the Institutional Review Board (IRB) of Eiken Chemical Co., Ltd. (No. 81–008 and No. 82-007), International University of Health and Welfare (18-Ifh-066) and Kouhoukai (No. 224 and No. 333), and informed consent was obtained from all participants. Of these, 60 samples (29 males, 31 females) were used for statistical analysis. Although individual ages were not available, most donors were adults in their 30s–70s, with ~70% in their 40s–60s. Individuals with a known history of cancer were excluded; comorbidity data were not available. Visibly lipemic or hemolyzed samples were excluded by visual inspection. Ten were pooled and used for the miRNA assay (**Figure 3**) to average out individual-specific characteristics and transient physiological variations. A sodium citrate-treated human plasma pool was purchased from Cosmo Bio Co., Ltd. (Japan) and used for lectin blot and immunoblot assays and as a reference standard for statistical data. Plasma was centrifuged at 10,000 × g for 10 min and filtered through a 0.45 µm pore-sized Surfactant Free Cellulose acetate membrane (Sartorius Stedim Biotech, Germany) to remove debris. The plasma was divided into small portions and stored at -80°C until use.

**Table 1:** Calculating of OAA1 bound to HM *N*-glycoproteins in CRC plasma or flow-through from the results of electrophoretic mobility shift assay (**Figure 1C**).

Lanes	Applied	Volume [µL]	Fluorescence intensity relative value*	Free OAA1 [µg]	Bound OAA1 [µg]
1	CRC plasma	7.5	0.15	0.375	2.125
2		5	0.38	0.95	1.55
3		2.5	0.88	2.2	0.3
4		1	1.11	2.775	-0.275
5	Flow-through	7.5	0.42	1.05	1.45
6		5	0.65	1.625	0.875
7		2.5	0.99	2.475	0.025
8		1	1.02	2.55	-0.05
9	BSA	5 μg	1.17	–	–
10	PBS	0	1	2.5	0

The linear approximation of free OAA1 in the CRC plasma is y = -0.3304x + 2.8172. *The relative fluorescence intensity was normalized to the fluorescence intensity observed when PBS was applied (lane 10).

### 2.2 Preparation of OAA1 and OAA1-immobilized column

A recombinant OAA variant, named OAA1, which contained two amino acid substitutions in OAA and an additional linker sequence at the C-terminal region for covalent immobilization, was prepared (unpublished data). OAA1 exhibited the same binding specificity as that of OAA. OAA1-covalently immobilized monolithic silica columns (OAA1-columns) were produced by Kyoto Monotech Co., Ltd. (Japan) (20). OAA1 chelating columns (OAA1-Ni-columns), in which Histidine-tagged (His-tagged) OAA1 is immobilized by chelating bond, were prepared as follows: His-tagged OAA1 solution (1 mg/mL; 200 µL) was applied to a Ni-NTA column (Kyoto Monotech Co., Ltd.) pre-washed using PBS (300 µL; FUJIFILM Wako Pure Chemical Corporation, Japan). After incubation for 5 min at 25°C, the flow-through solution was collected by centrifugation at 1,500 × g for 2 min. The columns were washed once with 300 µL of PBS and stored at 4°C in PBS. The amount of immobilized OAA1 was calculated from the difference in absorbance at 280 nm between the OAA1 and flow-through solutions from the column. The amount of immobilized OAA1 per OAA1-column was more than 60 µg.

### 2.3 Sodium dodecyl sulfate-polyacrylamide gel electrophoresis

SDS-PAGE was performed according to the Laemmli protocol (21). Each protein sample in sample loading buffer (62.5 mM Tris-HCl (pH 6.8), 2% SDS, 10% glycerol, 5% 2-mercaptoethanol, and 0.001% bromophenol blue) was boiled for 5 min before loading onto a 10–20% gradient gel (ATTO Corporation, Japan). Precision Plus Protein Dual Xtra Pre-stained Protein Standards (Bio-Rad Laboratories, Inc., United States) were used as protein molecular markers.

### 2.4 Labeling OAA1 with fluorescence

Tris(2-carboxyethyl)phosphine hydrochloride (TCEP-HCl, 100 mM; 1 µL, FUJIFILM Wako Pure Chemical Corporation) in degassed PBS was added to 100 µL of 100 µM OAA1 in degassed PBS (10 molar equivalents of TCEP-HCl to lectin) and incubated for 30 min at 37°C in the dark. Subsequently, 200 µL of 1 mM Alexa Fluor 488 C_5_ Maleimide (Thermo Fisher Scientific, United States) in degassed PBS (20 equiv) was added and incubated for 2 h at 25°C in the dark. The reaction was quenched by adding 20 µL of 100 mM glutathione (FUJIFILM Wako Pure Chemical Corporation) to degassed PBS (200 molar equivalents of glutathione to lectin) and incubating for 2 h at 25°C in the dark. The reaction mixture was applied to a Nanosep 3 K device (Pall Corporation, United States) and washed three times with 500 µL of PBS (14,000 × g, 10 min). The upper solution, containing the fluorophore-lectin conjugate, was collected. The concentration of fluorescently labeled OAA1 was determined to be approximately 50–100 µM using a BCA assay (Thermo Fisher Scientific). To confirm the fluorophore-lectin conjugates, 5 µL samples from each reaction step were separated using SDS-PAGE, OAA1 (16 kDa) was detected by Coomassie Brilliant Blue (CBB) staining, and fluorescence was scanned using FLA7000 (FUJIFILM Wako Pure Chemical Corporation) (**Figure 1A**).

### 2.5 Electrophoretic mobility shift assay

Sixty microliters of CRC plasma was added to an OAA1-column and centrifuged to collect the flow-through. Each microliter (7.5, 5.0, 2.5, or 1.0) of CRC plasma or the flow-through was mixed with 2.5 µg of the fluorescence-labeled OAA1 and incubated at 37°C for 30 min. Bovine serum albumin (BSA) (Nacalai Tesque, Inc., Japan) and PBS (7.5 µL) were used as negative controls. Each mixture was separated using SDS-PAGE, and fluorescence was scanned using an FLA7000. The fluorescence intensity of unbound OAA 1 (free OAA1) was measured using ImageJ 1.53 (National Institutes of Health, United States).

### 2.6 OAA1-captured material recovery from human plasma

The stored human plasma was centrifuged at 10,000 × g for 10 min to remove cell debris, and a portion (150 µL) of the supernatant was applied to a column. After allowing the column to stand for 5 min at 25°C, the flow-through plasma was collected by centrifugation at 1,500 × g for 2 min. The column was washed three times with 300 µL of 10xPBS (–) (FUJIFILM Wako Pure Chemical Corporation). To obtain OAA1-captures, elution buffer (100 µL) was applied to a column and collected by centrifugation at 400 × g for 2 min after standing for 5 min at 25°C. The elution buffers used for the immunoblotting analyses, nanoparticle counting, and miRNA assays were 0.4% SDS in PBS, 500 µM imidazole (FUJIFILM Wako Pure Chemical Corporation) in PBS, and QIAzol Lysis Reagent (QIAGEN, Germany), respectively.

### 2.7 Lectin blot analysis

Each plasma sample (1, 5, or 10 µg) diluted in PBS, 5 µg of purified thyroglobulin (Tg) (Merck KGaA, Germany), 10 µg of BSA, or 10 µL of eluted sample from a column was boiled with sample buffer for 5 min and loaded onto a 10–20% gradient gel. The gel was then transferred to Amersham Hybond LFP PVDF 0.2 (GE Healthcare Technologies Inc., United States). Membranes were blocked with 5% of BSA in TBS-T (10 mM Tris-HCl (pH 7.5), 150 mM NaCl, and 0.05% Tween 20). The membrane was incubated overnight with 2.5 µg/mL of the fluorescence-labeled OAA1 in TBS-T containing 5% of BSA at 4°C and fluorescent bands were scanned using the FLA7000.

### 2.8 Characterization of collected nanoparticles

#### 2.8.1 Immunoblot analysis

Each diluted plasma sample (1, 3, or 10 µg of total protein) in PBS and 20 or 5 µL of the eluted sample from a column with the sample loading buffer were boiled for 5 min and loaded onto a 12.5% SDS-PAGE gel. The proteins were then transferred to a nitrocellulose membrane (GE Healthcare Technologies, Inc.). The membranes were blocked with 3% BSA for CD9, blocking acetic acid for CD81, or 10% skim milk for albumin in TBS-T. The membrane was incubated with a primary antibody for 16 h at 4°C. After washing thrice with TBS-T for 15 min, the membrane was incubated with a secondary antibody for 1 h at 25°C. After washing thrice with TBS-T for 15 min, bands were detected using Immobilon Western Chemiluminescent HRP Substrate (Merck Millipore, United States) and X-ray films (GE Healthcare Technologies, Inc.).

#### 2.8.2 Particle size analysis and nanoparticle counting

Pooled CRC plasma (200 µL) was applied onto an OAA1-Ni-column, and OAA1-bound materials were coeluted with OAA1 ligand with imidazole (100 µL; 500 mM). The counting of particles in the OAA1-captures using a Tunable Resistive Pulse Sensor (qNano) (Izon Science, Ltd., New Zealand) was outsourced to Meiwafosis Co., Ltd. (Japan).

### 2.9 Antibodies

The antibodies and their dilutions were as follows: anti-human CD9 mouse monoclonal antibody (1:200, sc-59140; clone ALB6, Santa Cruz Biotechnology, Inc., United States), anti-CD81 mouse monoclonal antibody (1:200, sc-23962, clone 5A6, Santa Cruz Biotechnology), and anti-albumin rabbit polyclonal antibody (1:3,000, PAB028HU05, CLOUD-CLONE CORP., United States) for primary antibodies, HRP-conjugated anti-mouse IgG (1:1,000, P0447, Dako A/S, Denmark), and HRP-conjugated anti-rabbit IgG (1:1,000, NA934V, GE Healthcare Technologies Inc.) for secondary antibodies.

### 2.10 Isolation and purification of miRNA

Total RNA from 100 µL of column elution or 30 µL of each plasma sample was isolated using QIAzol Lysis Reagent. miRNAs were purified using a nucleospin miRNA plasma kit (MACHEREY–NAGEL GmbH & Co. KG, Germany), according to the prescribed protocol.

### 2.11 MicroRNA assay

The miRNA assay was performed as described previously (22). Purified miRNAs were reverse transcribed using the TaqMan MicroRNA Reverse Transcription Kit and miRNA-specific primers (Thermo Fisher Scientific). The miRNAs were quantified by Real-Time PCR (RT-qPCR) using a TaqMan miRNA assay kit and a 7500 Real-Time PCR system, according to the FAST protocol (Thermo Fisher Scientific). The TaqMan miRNA assay IDs and miRBase accession numbers for the miRNAs used in this study are listed in **Supplementary Table 1**. Synthesized cel-miR-39 (Norgen Biotek Corp., Canada) was spiked at 33 attomoles per sample as an external control. The cycle threshold (C_T_) values were determined using fixed threshold settings. Relative expression levels of the target miRNAs were normalized to cel-miR-39 using the ΔΔC_T_ method (23).

### 2.12 Statistical analysis

An experimental flowchart is presented in **Supplementary Figure 2**. There was no appropriate internal control for the relative quantification of circulating miRNA expression levels. Therefore, it is standard practice to either spike synthetic miRNA from non-human sources as an external control or to use the average value of three miRNAs with relatively low variability as an internal control (24). We used two normalization methods. External normalization: the C_T_ value of miRNA by RT-qPCR was normalized using spiked-in cel-miR-39, and the delta-delta C_T_ value was calculated by subtracting the ΔC_T_ value of a standard reference (commercial pooled healthy plasma) from the ΔC_T_ value, giving a ΔCT_ext_ value. Internal normalization: The C_T_ value was normalized using the average C_T_ value (CT_int_) of these three miRNAs (miR-93-5p, miR-192-5p, and miR-502-5p), which were chosen as internal control miRNAs in this study (Results section 3.5), yielding a ΔCT_int_ value. The ΔCT_ext_ and ΔCT_int_ values were used for statistical processing. Logistic regression analysis and evaluation of indices for logistic regression models were performed using R version 3.5.2 (R Core Team, R Foundation for Statistical Computing, Vienna, Austria). Predictor variables were selected using the backward-forward stepwise method based on the Akaike information criterion (AIC). The net reclassification improvement (NRI) and Integrated discrimination improvement (IDI) were calculated using R (25). The positive predictive value (PPV) was calculated using a previously described method (26). Statistical significance was set at *p* < 0.05 significant.

## 3 Results

### 3.1 OAA1 bound to high-mannose *N*-glycans in human plasma

OAA1 was labeled with Alexa-488 (**Figure 1A**). Lectin blot analysis using labeled OAA1 demonstrated that OAA1 binds to certain proteins in human plasma (**Figure 1B**), which are likely high-mannose *N*-glycan linked glycoproteins (HM *N*-glycoproteins), given OAA’s high binding specificity of OAA (6). The labeled OAA1 bound to thyroglobulin (Tg), a ~660 kDa precursor of thyroid hormones containing HM *N*-glycans (27), but did not bind to bovine serum albumin (BSA) or serum albumin in human plasma, which are not HM *N*-glycosylated (**Figure 1B**). Immunoblot analysis detected low levels of serum albumin in the blank column, likely due to non-specific binding (**Figure 2A**).

After mixing fluorescently labeled OAA1 with plasma and performing electrophoresis, the band corresponding to unbound OAA1 (free OAA1) diminished. This suggests that OAA1 binds to HM *N*-glycoproteins in plasma, resulting in a decrease in free OAA1 levels (**Figure 1C**). The amount of bound OAA1 was calculated based on the decrease in the signal intensity of free OAA1 (**Table 1**). To confirm whether the OAA1-column could capture HM *N*-glycan in the plasma, we performed the same electrophoretic mobility shift assay with the flow-through of the OAA1-column (see Materials and Methods for details). The bound OAA1 in the flow-through was lower than that in the plasma (**Table 1**), suggesting that the OAA1-column could capture some HM *N*-glycan in the plasma. The calculated plasma volume required for all 2.5 µg of OAA1 to bind is 8.53 µL (the linear approximation of free OAA1 in the CRC plasma is y = -0.3304x + 2.8172). Therefore, the amount of OAA1 needed to bind all HM *N*-glycans contained in the applied 60 µL of plasma is 17.6 µg. Despite sufficient OAA1 immobilization on the OAA1-column, more than half of the OAA1-binding components remained in the flow-through (**Figure 1C**). This suggests that there may be a limitation to the binding protocol using the OAA1 immobilized spin column device.

### 3.2 OAA1-captured EVs in human plasma

Immunoblotting assay demonstrated that CD81, an EV marker, was detected in OAA1-captured CRC plasma, but not in healthy plasma (**Figure 2A**). Nanoparticle analysis of OAA1-captures revealed that particles 50–150 nm in diameter constituted 87.4% of the counted nanoparticles (40–1,300 nm) (**Figure 2B**). Given that OAA1 specifically binds to HM *N*-glycans (**Figures 2B**, **3A**) (7), OAA1-captured EVs are likely to be HM *N*-glycosylated.

### 3.3 MiRNA profiling of OAA1-captured EVs derived from patients with CRC stage II-IV

Comprehensive profiling of miRNAs in OAA1-captured from pooled CRC plasma or pooled healthy plasma showed that 17 miRNAs were upregulated, 13 miRNAs folded down, and 12 miRNAs were stable in CRC plasma compared to healthy controls (**Supplementary Table 2**). Based on these results and several previous reports (10–14, 28, 29), we selected 12 miRNAs and performed quantitative Reverse Transcription Polymerase Chain Reaction (RT-qPCR) analysis of miRNA in OAA1-captures from CRC and healthy plasma (**Figure 3**). In OAA1-captures from the pooled plasma of 10 patients with CRC (stage II to IV), miR-21-5p, miR-130a-3p, miR-146a-5p, miR-126, miR-15b-5p, miR-423-3p, miR-23a-3p, and miR-199-3p were significantly increased (*p* < 0.001), while miR-122-5p and miR-192-5p were significantly decreased (*p* < 0.001) compared with pooled healthy plasma. There were no significant differences in miR-93-5p (*p* = 0.10) and miR-502-5p (*p* = 0.49) levels between CRC and healthy plasma samples (**Figure 3**).

Furthermore, the mixture experiments showed that the fluctuations of some miRNAs in OAA1-captured samples were dependent on the CRC plasma concentration (**Supplementary Figure 1**). The relative values of each miRNA captured by the column indicated that miR-21-5p, miR-130a-3p, miR-146a-5p, miR-15b-5p, miR-126, and miR-423-3p increased, whereas miR-122-5p decreased depending on the CRC plasma concentration, but not miR-93-5p or miR-199a-5p. If OAA1 captured EVs unrelated to cancer, or if there were any inhibitors or activators affecting the capture ability of OAA1 in plasma, the fluctuations of the miRNAs in OAA1-captures would not have shown a mixing ratio-dependent manner. These data suggest that the OAA1-column captured circulating miRNAs derived from patients with CRC and that there were no inhibitors or activators affecting the capture ability of OAA1 in the plasma.

### 3.4 Selection of OAA1-captured circulating miRNAs as biomarkers for early-stage colorectal cancer

**Figure 4:**
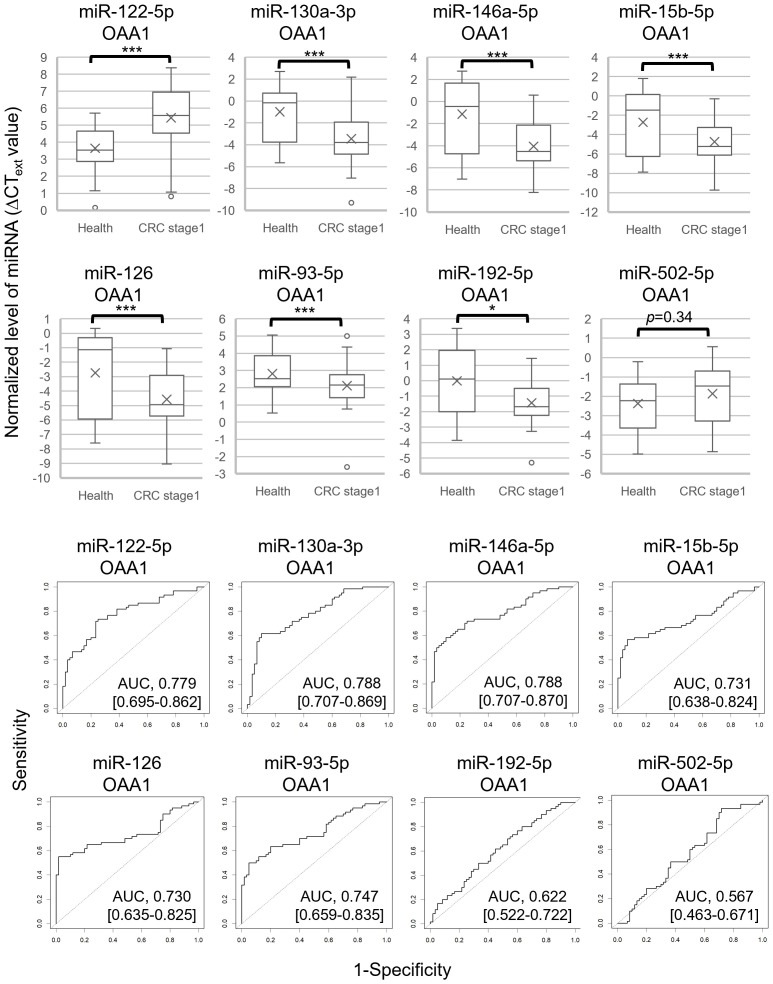
Eight miRNAs in OAA1-captures from plasma of 60 healthy controls and 60 patients with CRC stage I. The values were normalized to cel-miR-39 as an external control and then to the average ΔC_T_ value of the reference sample (ΔCT_ext_). **(A)** Each level of 8 miRNAs in OAA1-captures is shown in ΔCT_ext_ value. Each value of mean (x), error (SD), median (black lines), and 25th–75th percentiles (boxes) is detailed in **Supplementary Table 3**. Statistical significance was determined using Welch’s t-test or Mann–Whitney U test; normality was determined using the Shapiro–Wilk normality test (*p < 0.05, ***p < 0.001). **(B)** ROC curves for detecting stage I CRC using 8 miRNAs. AUC, area under the curve. The 95% confidence interval is shown in **Supplementary Table 3**.

To select miRNA biomarker candidates for early stage CRC, we analyzed miRNA levels in the OAA1-captures from the plasma of 60 patients with CRC stage I and 60 healthy controls (**Figure 4A**, **Supplementary Table 3**). Similar to the results obtained using the pooled plasma of patients with CRC (stage II to IV) and healthy controls (**Figure 3**), there were statistically significant differences between patients with stage I CRC and healthy controls in miR-122-5p, miR-130a-3p, miR-146a-5p, miR-15b-5p, miR-126 (*p* < 0.001), and miR-192-5p (*p* < 0.05), but not miR-502-5p (*p* = 0.34). Additionally, miR-93-5p expression differed significantly (*p* < 0.001). Receiver operating characteristic (ROC) curve analysis indicated that miR-122-5p (area under the curve (AUC) = 0.779), miR-130-3p (AUC = 0.788), miR-146a-5p (AUC = 0.788), miR-15b-5p (AUC = 0.731), miR-126 (AUC = 0.730), and miR-93-5p (AUC = 0.747) were good biomarker candidates (**Figure 4B**).

### 3.5 Selection of three miRNAs as internal controls for analysis of OAA1-captured circulating miRNAs in patients with CRC

The actual measurements of liquid biopsies are influenced by multiple pre-analytical factors, including technical issues related to collection and examination, as well as variations among column lots. Therefore, selecting an internal control that normalizes all variables is crucial for the relative evaluation of circulating miRNAs. In this study, we selected three miRNAs as internal control candidates: miR-93-5p, miR-192-5p, and miR-502-5p, based on the following statistical criteria: 1) the standard deviation (SD) of the ΔCT_ext_ values for these miRNAs was lower than that of the others (**Supplementary Table 3**); 2) the AUC values for miR-192-5p (0.622) and miR-502-5p (0.567) were close to 0.5 (**Figure 4B**); 3) no significant difference was observed in miR-93-5p (*p* = 0.10) and miR-502-5p (*p* = 0.49) between pooled CRC plasma and pooled healthy plasma (**Figure 3**, **Supplementary Figure 1**).

**Figure 5:**
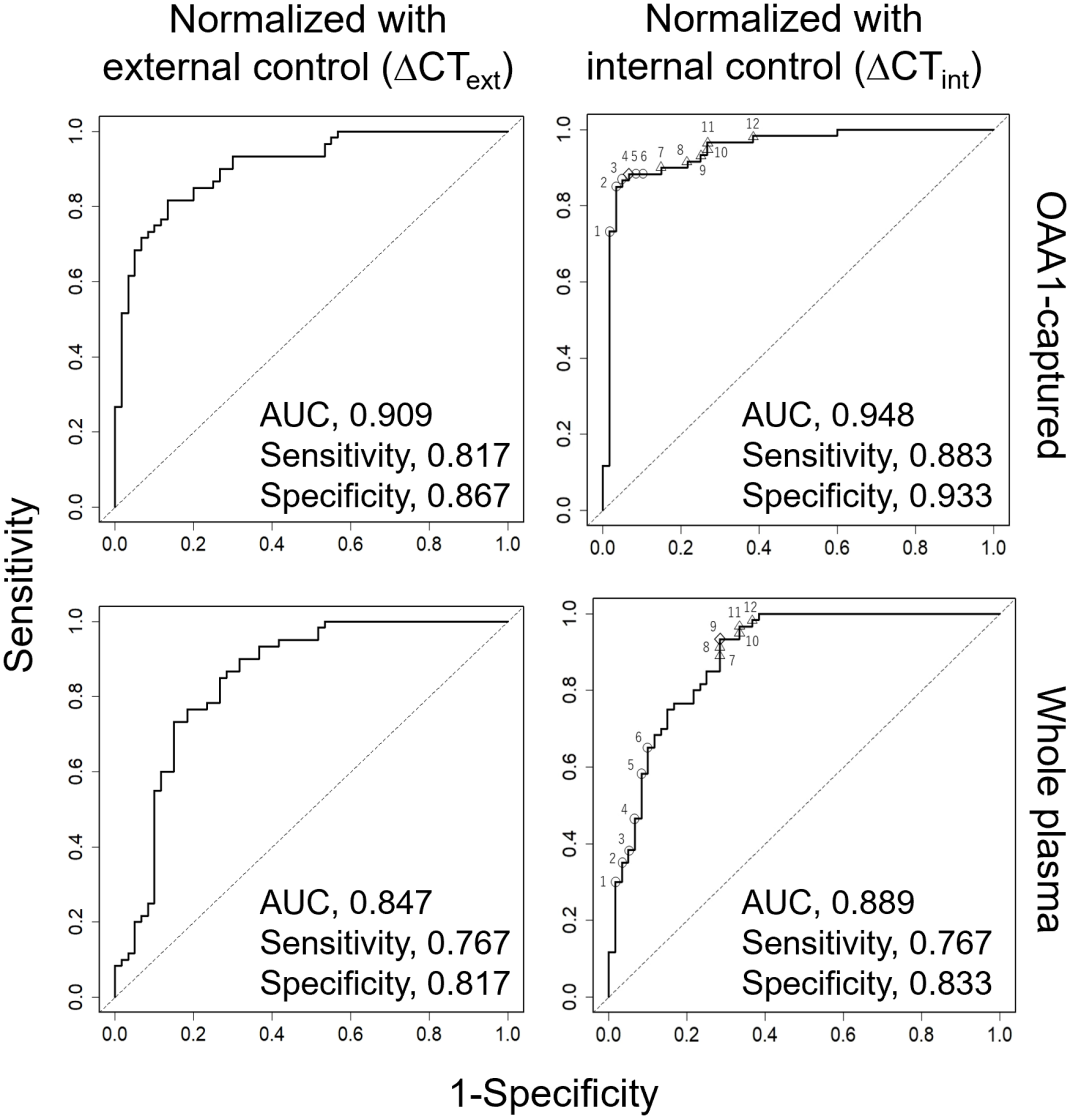
ROC curve analysis for detection of CRC using five miRNAs (miR-122-5p, miR-130a-3p, miR-146a-5p, miR-15b-5p, miR-126) from 60 healthy and 60 patients with stage I CRC. Each miRNA was eluted from OAA1-captured (upper row) or whole plasma (bottom row). Data were normalized to cel-miR-39 as an external control and then to the average ΔC_T_ value of the reference sample (ΔCT_ext_, left columns), or normalized to the average C_T_ value of three miRNAs (miR-93-5p, miR-192-5p, and miR-502-5p) as an internal control (ΔCT_int_, right columns). Statistical data are detailed in **Table 2**. Statistical data of numbered plots in the right panels are shown in **Table 3**. AUC, area under the curve.

The average C_T_ value of the three miRNAs was used as an internal control in the subsequent statistical analysis involving 60 patients with CRC stage I and 60 healthy controls (**Figure 5**, **Tables 2**, **3**, **Supplementary Tables 4**, **5**). The use of this internal control resulted in lower SDs for each explanatory variable (**Supplementary Table 3**) and improved the logistic model’s ability to separate CRC stage I from the controls compared with the external control (**Supplementary Tables 4**, **5**). The Nagelkerke R^2^ ratio also indicated that the model using internal controls fit the logistic regression data better than the model using external controls (**Table 2**). Consequently, the ROC curve analysis normalized to the internal control demonstrated a higher potential for cancer separation than normalization to the external control (right columns in **Figure 5**, **Table 2**).

**Table 2:** Statistics for model improvement.

Normalization	External control (ΔCT_ext_)		Internal control (ΔCT_int_)	
		*p*-value		*p*-value
Patients with stage I CRC (*n*)	60		60	
Healthy controls (*n*)	60		60	
Hosmer–Lemeshow goodness of fit				
OAA-captured	6.380	0.6052	21.797	0.0053
whole plasma	11.200	0.1914	8.542	0.3824
Nagelkerke R^2^ (ratio)				
OAA-captured	0.624		0.725	
whole plasma	0.478		0.584	
NRI (Continuous) [95% CI] (ratio)	0.867 [0.544–1.189]	< 0.01	0.867 [0.546–1.187]	< 0.01
IDI [95% CI] (ratio)	0.130 [0.051–0.210]	< 0.01	0.165 [0.086–0.245]	< 0.01
AUC [95% CI] (ratio)				
difference	0.062 [0.040–0.084]	0.0742	0.060 [0.042–0.078]	< 0.05
OAA-captured	0.909 [0.858–0.960]		0.948 [0.908–0.989]	
whole plasma	0.847 [0.774–0.920]		0.889 [0.830–0.947]	
Cut off point optimized by Youden’s index				
OAA-captured	0.5328		0.5993	
whole plasma	0.5894		0.5776	
Sensitivity (ratio)				
OAA-captured	0.817		0.883	
whole plasma	0.767		0.767	
Specificity (ratio)				
OAA-captured	0.867		0.933	
whole plasma	0.817		0.833	
Positive predictive value (ratio)				
OAA-captured	0.860		0.930	
whole plasma	0.807		0.821	
Negative predictive value (ratio)				
OAA-captured	0.825		0.889	
whole plasma	0.778		0.781	

CRC, colorectal cancer; NRI, net reclassification improvement; IDI, integrated discrimination improvement; AUC, area under the curve; CI, confidence interval. ΔCT_ext_ = Patient ΔC_T_ (C_TmiR-XX_ – C_Tcel-miR-39_) – Reference Standard ΔC_T_ (C_TmiR-XX_ – C_Tcel-miR-39_). ΔCT_int_ = C_TmiR-XX_ – average C_T_ value of miR-93-5p, miR-192-5p and miR-502-5p.

**Table 3:** Positive predictive value (ratio) of twelve cut-off points.

OAA1-captured (ΔCT_int_)						Whole plasma (ΔCT_int_)					
	SE	SP	Accuracy	PPV (P = 0.002)	PPV (P = 0.035)		SE	SP	Accuracy	PPV (P = 0.002)	PPV (P = 0.035)
1	0.733	0.983	0.858	0.081	0.615	1	0.300	0.983	0.642	0.035	0.395
2	0.850	0.967	0.908	0.049	0.480	2	0.350	0.967	0.658	0.021	0.276
3	0.867	0.950	0.908	0.034	0.386	3	0.383	0.950	0.667	0.015	0.218
**4**	**0.883**	**0.933**	**0.908**	**0.026**	**0.325**	4	0.467	0.933	0.700	0.014	0.202
5	0.883	0.917	0.900	0.021	0.278	5	0.583	0.917	0.750	0.014	0.202
6	0.883	0.900	0.892	0.017	0.243	6	0.650	0.900	0.775	0.013	0.191
7	0.900	0.850	0.875	0.012	0.179	7	0.900	0.717	0.808	0.006	0.103
8	0.917	0.783	0.850	0.008	0.133	8	0.917	0.717	0.817	0.006	0.105
9	0.933	0.750	0.842	0.007	0.119	**9**	**0.933**	**0.717**	**0.825**	**0.007**	**0.107**
10	0.950	0.733	0.842	0.007	0.114	10	0.950	0.667	0.808	0.006	0.094
11	0.967	0.733	0.850	0.007	0.116	11	0.967	0.667	0.817	0.006	0.095
12	0.983	0.617	0.800	0.005	0.085	12	0.983	0.633	0.808	0.005	0.089

Numbered plots in ROC in the right panels of **Figure 5**. The cut-off point optimized by Youden’s index (bold). The values of prevalence (P) are according to Japanese mass screening data in 1991 (30). SE, sensitivity; SP, specificity; P, prevalence; PPV, positive predictive value, PPV = P*SE/(P*SE + (1–P) (1–SE)). ΔCT_int_ = C_TmiR-XX_ – average C_T_ value of miR-93-5p, miR-192-5p and miR-502-5p.

### 3.6 Validity of OAA1-captured circulating miRNAs in human plasma

We conducted a miRNA analysis of whole plasma samples from 60 patients with stage I CRC and 60 healthy controls. Logistic regression analysis using miRNAs in OAA1-captured EVs demonstrated superior statistical results compared with miRNAs in whole plasma (**Figure 5**, **Table 2**, **Supplementary Table 5**). Additionally, the predictive capability of the multivariate model with OAA1-captured circulating miRNA biomarkers significantly improved, as indicated by two metrics for comparative evaluation of the two AUCs: net reclassification improvement (NRI, *p* < 0.01) and integrated discrimination improvement (IDI, *p* < 0.01)(**Table 2**) (25). ROC curve analysis using miRNAs in OAA1-captured EVs normalized to the internal control showed the highest potential for cancer separation ability (AUC, 0.948; sensitivity, 0.883; specificity, 0.933) (right upper panel in **Figure 5**, **Supplementary Table 5**). The highest Nagelkerke R^2^ ratio (0.725) further supported this model as the best among the four for explaining the data, and the Hosmer–Lemeshow goodness-of-fit test indicated a good fit (*p* > 0.05) (**Table 2**).

## 4 Discussion

Glycosylation is a crucial cellular mechanism that regulates various physiological and pathological processes. Glycosylation alterations and abnormalities in cancer cells have garnered significant attention because of their potential in the diagnosis and treatment of neoplastic diseases (31, 32). Elevated levels of high mannose *N*-glycosylation have been observed in several cancers, including ovarian (33, 34), breast (35), and prostate (36, 37) cancer. Furthermore, specific changes in glycosylation are involved in hematologic malignancies (38), neurodegenerative diseases such as Alzheimer’s disease (59), and infectious diseases (39). Additionally, age-related changes in glycosylation are known to contribute to the onset of diseases such as cancer and dementia (30). Glycosylation plays crucial roles in the diagnosis and treatment of these diseases.

Based on our results, the OAA1-column captured EVs from human blood, which are expected to contain HM *N*-glycans. EVs retain several features of their parent cells, including miRNAs (10). We hypothesized that the OAA1-column could capture miRNAs in EVs derived from tumor cells, which might reflect the origin and function of these tumor cells. Therefore, we anticipated that miRNAs in OAA1-captured EVs could provide more sensitive detection of subtle tumor characteristics than whole circulating miRNAs. Liquid biopsy is expected to be a relevant approach for early detection through annual medical checks and postoperative monitoring owing to its low invasiveness and rapid results. With advancements in detection technologies and the discovery of new biomarkers, their development has become remarkable. However, body fluids contain a wide range of biological information released from various cells, and simply increasing the detection sensitivity increases the risk of false positives. Purification processes are effective in avoiding false positives and improving the detection accuracy. In this study, we propose a pretreatment method using lectins. Lectins bind to glycans with high specificity. By selectively collecting glycans that appear as signs of disease using lectins and detecting the target, the detection accuracy can be improved. Lectins are easier to process and handle than antibodies and can be used in simple purification devices, such as spin columns. A pretreatment system using lectins has the potential to improve the convenience and accuracy of liquid biopsies.

In Japan, annual fecal occult blood testing (FOBT) is recommended as the primary screening method for CRC (40). However, the follow-up colonoscopy rate among FOBT-positive individuals remains below 50%, largely due to the low positive predictive value (PPV) (26) of FOBT (typically <10%) (41), the asymptomatic nature of early-stage CRC, and the physical, psychological, and financial burdens associated with colonoscopy (42, 43). These limitations highlight the urgent need for a more accessible and accurate secondary screening strategy. Although circulating miRNAs have been extensively studied as potential CRC biomarkers (12–14), their PPV remains limited in low-prevalence populations, even when sensitivity and AUC exceed 95% (44). For example, in a large-scale Japanese screening program (1988), the PPV of fecal immunochemical testing (FIT) was only 0.035 for early-stage CRC (45). As shown in **Table 3**, our OAA1-captured EV miRNA assay significantly improved PPV to 0.615 when used as a secondary screen following FIT, far surpassing the performance of FIT alone or miRNA assays using whole plasma. These findings support the clinical utility of our assay as a practical and effective secondary screening tool. Importantly, it may serve as a confirmatory step before recommending colonoscopy, helping to identify individuals who are more likely to benefit from further invasive testing. By improving the accuracy of secondary screening, this approach has the potential to reduce unnecessary colonoscopies and increase follow-up compliance among FIT-positive individuals.

In the present study, we confirmed the presence of OAA1-captured EVs in the plasma of patients with CRC (**Figures 2**). Our previous data indicated that OAA1-captured EVs released from cancer cells, but not from normal cell lines (1). Collectively, these findings suggest that OAA1-captured EVs from CRC plasma may be tumor-derived EVs released into the blood from tumor cells. However, the OAA1-column also captured EVs from healthy plasma, suggesting that HM *N*-glycosylated EVs may be released not only from tumor cells, but also from other cells with HM *N*-glycosylated membrane proteins (46). HM *N*-glycans would exist in various states in the plasma, such as on the surface of nanoparticles (e.g., EVs and lipoproteins), fragments of cells, blood proteins, or free molecules. We verified that some plasma proteins bound to OAA1 (**Figures 1B, C**). Apolipoprotein B, complement C3 alpha-2-macrogloblin and other blood proteins were confirmed in OAA1-captures by Nano-LC/MS/MS analysis (unpublished data), indicating that these proteins were HM *N*-glycosylated. Immunoblot analyses showed that the CD81-positive band, an EV marker, was increased in the CRC plasma (**Figure 2A**). It is known that EV secretion by cancer cells is higher than that by normal cells (47), and that the amount of circulating EVs in cancer patients is elevated (48). Our nanoparticle measurements confirmed that EV particles were approximately 10 times more abundant than in healthy plasma (data not shown). These results suggest that HM-EVs may increase in patients with CRC and potentially originate from CRC cells.

To further investigate the origin of OAA1-captured EVs, future glycoproteomic profiling may offer a promising strategy for distinguishing tumor-derived vesicles from those released by non-malignant cells. Cancer-related EVs are known to exhibit distinct glycosylation patterns which are rarely observed in EVs from healthy tissues (49). These glycan features could serve as molecular signatures of malignancy. Advanced analytical techniques—including lectin-based enrichment, mass spectrometry, and glycan-specific antibodies—may enable the identification of such signatures in OAA1-captured EVs. Furthermore, integrating glycoproteomic data with genetic approaches such as single EV sequencing could facilitate precise tracing of EV origin and provide deeper insights into their biological roles. These combined strategies may enhance the specificity of EV-based biomarkers and contribute to the development of non-invasive diagnostic tools for cancer.

Circulating non-EV-miRNAs are important for homeostasis and disease development (50). Further studies are required to establish the significance of OAA1-captured circulating miRNAs as potential biomarkers. To identify CRC-specific miRNA biomarkers, we initially used pooled plasma samples from healthy controls in the comprehensive screening phase (**Figure 3**). Pooling was intended to reduce individual variability and transient physiological fluctuations, thereby minimizing non-CRC-related noise and enhancing the detection of CRC-specific signals. However, we acknowledge that this approach may obscure subtle inter-individual differences. To address this, complementary analyses using individual samples were conducted to validate the findings and refine the candidate miRNAs (**Figures 4**, **5**). This two-step strategy—broad screening with pooled samples followed by individual-level validation—proved effective for biomarker discovery in liquid biopsy research.

Considering that OAA1 specifically captures HM-EVs (1), OAA1-captured EVs increased in CRC plasma compared to healthy plasma (**Figure 2A**), and that changes depend on CRC plasma in mixed experiments (**Supplementary Figure 1**), we hypothesized that the changes observed in miRNAs captured by OAA1 might reflect circulating EVs derived from CRC tumor cells, which might be encased in HM *N*-glycosylated membranes.

Given that the detection of spiked-in miRNAs remained unaffected, the influence of co-captured HM-glycoproteins on miRNA analysis appears negligible. In contrast, the OAA1-column effectively removed abundant plasma proteins such as albumin, which facilitated more efficient miRNA extraction. Importantly, all 12 miRNAs differentially expressed between CRC and healthy plasma shown in **Figure 3** were identified as EV-associated (51, 52), suggesting that the OAA1-column reduces non-EV miRNA interference and enriches for tumor-derived EV-miRNAs. This selective enrichment likely contributed to the improved diagnostic performance observed in OAA1-captured fractions.

EV-associated miRNAs reflect the molecular characteristics of their cells of origin and are widely studied as disease biomarkers (10–14). Although the five miRNAs identified here have not been previously reported as CRC-specific, all are known to circulate in EVs. In contrast, well-known CRC-related miRNAs such as miR-147b (53) and the miR-200 family (54), commonly found in tumor tissues, were not detected in our profiling (**Supplementary Table 2**). We also excluded miR-21-5p and miR-23a-5p—well-known oncomiRs (55, 56)—despite their significant elevation in CRC plasma (**Figure 3**), due to their variability in non-cancerous conditions (57).

MiRNAs regulate the expression of epigenetic genes. In this study, five miRNAs were selected as biomarkers for identifying early stage CRC, such as stage I. Gene target analyses of these miRNAs were performed based on database searches (see **Supplementary Methods** for details). **Supplementary Table 6** lists the target gene candidates related to the CRC pathway. These genes play key roles in various pathways involved in the development of CRC, functioning as transcription factors, receptors, and accessory proteins of enzymes. Although it remains unclear whether miRNAs in circulating EVs are directly related to cancer progression, the miRNA profiles of circulating EVs may reflect the condition of the body or cells owing to illness, progression, and prognosis (58). These data support the high AUC values and the use of circulating HM EVs as new CRC biomarkers.

## 5 Conclusion

OAA-immobilized columns may have the potential to collect tumor-derived EVs from plasma. Analysis of the miRNAs extracted from these EVs demonstrated promising AUC values, suggesting their potential utility in the early diagnosis of CRC.

## Data Availability

The datasets presented in this study can be found in online repositories. The names of the repository/repositories and accession number(s) can be found in the article/**Supplementary Material**.

## References

[1] Yamamoto M Harada Y Suzuki T Fukushige T Yamakuchi M Kanekura T. Application of high-mannose-type glycan-specific lectin from Oscillatoria Agardhii for affinity isolation of tumor-derived extracellular vesicles. Anal Biochem. (2019) 580:21–9. doi: 10.1016/j.ab.2019.06.001 31173727

[2] Sethi MK Kim H Park CK Baker MS Paik YK Packer NH. In-depth N-glycome profiling of paired colorectal cancer and non-tumorigenic tissues reveals cancer-, stage- and EGFR-specific protein N-glycosylation. Glycobiology. (2015) 25:1064–78. doi: 10.1093/glycob/cwv042 26085185

[3] Adamczyk B Tharmalingam T Rudd PM. Glycans as cancer biomarkers. Biochim Biophys Acta. (2012) 1820:1347–53. doi: 10.1016/j.bbagen.2011.12.001 22178561

[4] Boyaval F Dalebout H Van Zeijl R Wang W Fariña-Sarasqueta A Lageveen-Kammeijer GSM. High-mannose N-glycans as Malignant progression markers in early-stage colorectal cancer. Cancers (Basel). (2022) 14(6):1552. doi: 10.3390/cancers14061552 35326703 PMC8945895

[5] Sato Y Murakami M Miyazawa K Hori K. Purification and characterization of a novel lectin from a freshwater cyanobacterium, Oscillatoria agardhii. Comp Biochem Physiol B Biochem Mol Biol. (2000) 125:169–77. doi: 10.1016/S0305-0491(99)00164-9 10817903

[6] Sato Y Okuyama S Hori K. Primary structure and carbohydrate binding specificity of a potent anti-HIV lectin isolated from the filamentous cyanobacterium Oscillatoria agardhii. J Biol Chem. (2007) 282:11021–9. doi: 10.1074/jbc.M701252200 17314091

[7] Sato T Hori K. Cloning, expression, and characterization of a novel anti-HIV lectin from the cultured cyanobacterium, Oscillatoria agardhii. Fisheries Science. (2009) 75:743–53. doi: 10.1007/s12562-009-0074-4

[8] Lee RC Feinbaum RL Ambros V. The C. elegans heterochronic gene lin-4 encodes small RNAs with antisense complementarity to lin-14. Cell. (1993) 75:843–54. doi: 10.1016/0092-8674(93)90529-Y 8252621

[9] Valadi H Ekström K Bossios A Sjöstrand M Lee JJ Lötvall JO. Exosome-mediated transfer of mRNAs and microRNAs is a novel mechanism of genetic exchange between cells. Nat Cell Biol. (2007) 9:654–9. doi: 10.1038/ncb1596 17486113

[10] Shah R Tushar Patel T Freedman JE. Circulating extracellular vesicles in human disease. N Engl J Med. (2018) 379:958–66. doi: 10.1056/NEJMra1704286 30184457

[11] Kosaka N Iguchi H Ochiya T. Circulating microRNA in body fluid: a new potential biomarker for cancer diagnosis and prognosis. Cancer Sci. (2010) 101:2087–92. doi: 10.1111/j.1349-7006.2010.01650.x PMC11159200 20624164

[12] Toiyama Y Okugawa Y Fleshman J Richard Boland C Goel A. MicroRNAs as potential liquid biopsy biomarkers in colorectal cancer: A systematic review. Biochim Biophys Acta Rev Cancer. (2018) 1870:274–82. doi: 10.1016/j.bbcan.2018.05.006 PMC7286572 29852194

[13] Ogata-Kawata H Izumiya M Kurioka D Honma Y Yamada Y Furuta K. Circulating exosomal microRNAs as biomarkers of colon cancer. PloS One. (2014) 9:e92921. doi: 10.1371/journal.pone.0092921 24705249 PMC3976275

[14] Datta B Paul D Dey T Pal S Rakshit T. Importance of extracellular vesicle derived RNAs as critical colorectal cancer biomarkers. ACS Bio Med Chem Au. (2022) 2:222–35. doi: 10.1021/acsbiomedchemau.1c00043 PMC10114864 37101571

[15] International Agency for Research on Cancer (IARC). Global cancer observatory. Lyon, France: IARC (2025). Available online at: https://gco.iarc.fr/en (accessed April 1, 2025).

[16] Services CfCCaI. Projected cancer deathes in 2022. Tokyo, Japan: Foundation for Promotion of Cancer Research (2022).

[17] Research FfPoC. Cancer Statistics In Japan-2023–2023 [93] . Available online at: https://ganjoho.jp/public/qa_links/report/statistics/2023_jp.html (Accessed April 1, 2025).

[18] Hospital-based Cancer Registry: 5-year Survival at the Designated Cancer Care Hospitals in 2013-2014. Tokyo, Japan: National Cancer Center Japan, Center for Cancer Control and Information Services (2023).

[19] Rectum JSfCotCa. Japanese classification of Colorectal, Appendiceal, and Anal Carcinoma. Ninth ed. Tokyo, Japan: KANEHARA & CO., LTD (2018). p. 132.

[20] Japanese Patent Application Disclosure 2000-119300. Tokyo, Japan: Inventor of the patent is Masahiro Iwakura (2000).

[21] Laemmli UK. Cleavage of structural proteins during the assembly of the head of bacteriophage T4. Nature. (1970) 227:680–5. doi: 10.1038/227680a0 5432063

[22] Schmittgen TD Lee EJ Jiang J Sarkar A Yang L Elton TS. Real-time PCR quantification of precursor and mature microRNA. Methods. (2008) 44:31–8. doi: 10.1016/j.ymeth.2007.09.006 PMC2663046 18158130

[23] Livak KJ Schmittgen TD. Analysis of relative gene expression data using real-time quantitative PCR and the 2(-Delta Delta C(T)) Method. Methods. (2001) 25:402–8. doi: 10.1006/meth.2001.1262 11846609

[24] Hu Z Dong J Wang LE Ma H Liu J Zhao Y. Serum microRNA profiling and breast cancer risk: the use of miR-484/191 as endogenous controls. Carcinogenesis. (2012) 33:828–34. doi: 10.1093/carcin/bgs030 22298638

[25] Pickering JW Endre ZH. New metrics for assessing diagnostic potential of candidate biomarkers. Clin J Am Soc Nephrol. (2012) 7:1355–64. doi: 10.2215/CJN.09590911 22679181

[26] Baeyens JP Serrien B Goossens M Clijsen R. Questioning the "SPIN and SNOUT" rule in clinical testing. Arch Physiother. (2019) 9:4. doi: 10.1186/s40945-019-0056-5 30891312 PMC6407254

[27] Ronin C Fenouillet E Hovsepian S Fayet G Fournet B. Regulation of thyroglobulin glycosylation. A comparative study of the thyroglobulins from porcine thyroid glands and follicles in serum-free culture. J Biol Chem. (1986) 261:7287–93. doi: 10.1016/S0021-9258(17)38388-6 2423517

[28] Igaz I Igaz P. Tumor surveillance by circulating microRNAs: a hypothesis. Cell Mol Life Sci. (2014) 71:4081–7. doi: 10.1007/s00018-014-1682-4 PMC4194016 25037157

[29] Yoshikawa Y Fukunaga M Takahashi J Shimizu D Masuda T Mizushima T. Identification of the minimum combination of serum microRNAs to predict the recurrence of colorectal cancer cases. Ann Surg Oncol. (2023) 30:233–43. doi: 10.1245/s10434-022-12355-w PMC9726799 36175711

[30] Zhang W Chen T Zhao H Ren S. Glycosylation in aging and neurodegenerative diseases. Acta Biochim Biophys Sin (Shanghai). (2024) 56:1208–20. doi: 10.3724/abbs.2024136 PMC11466714 39225075

[31] Stowell SR Ju T Cummings RD. Protein glycosylation in cancer. Annu Rev Pathol. (2015) 10:473–510. doi: 10.1146/annurev-pathol-012414-040438 25621663 PMC4396820

[32] Pinho SS Reis CA. Glycosylation in cancer: mechanisms and clinical implications. Nat Rev Cancer. (2015) 15:540–55. doi: 10.1038/nrc3982 26289314

[33] Anugraham M Jacob F Nixdorf S Everest-Dass AV Heinzelmann-Schwarz V Packer NH. Specific glycosylation of membrane proteins in epithelial ovarian cancer cell lines: glycan structures reflect gene expression and DNA methylation status. Mol Cell Proteomics. (2014) 13:2213–32. doi: 10.1074/mcp.M113.037085 PMC4159645 24855066

[34] Alley WR Vasseur JA Goetz JA Svoboda M Mann BF Matei DE. N-linked glycan structures and their expressions change in the blood sera of ovarian cancer patients. J Proteome Res. (2012) 11:2282–300. doi: 10.1021/pr201070k PMC3321095 22304416

[35] Liu X Nie H Zhang Y Yao Y Maitikabili A Qu Y. Cell surface-specific N-glycan profiling in breast cancer. PloS One. (2013) 8:e72704. doi: 10.1371/journal.pone.0072704 24009699 PMC3751845

[36] Shah P Wang X Yang W Toghi Eshghi S Sun S Hoti N. Integrated proteomic and glycoproteomic analyses of prostate cancer cells reveal glycoprotein alteration in protein abundance and glycosylation. Mol Cell Proteomics. (2015) 14:2753–63. doi: 10.1074/mcp.M115.047928 PMC4597149 26256267

[37] Drake RR Jones EE Powers TW Nyalwidhe JO. Altered glycosylation in prostate cancer. Adv Cancer Res. (2015) 126:345–82. doi: 10.1016/bs.acr.2014.12.001 25727153

[38] Su H Wang M Pang X Guan F Li X Cheng Y. When glycosylation meets blood cells: A glance of the aberrant glycosylation in hematological Malignancies. Rev Physiol Biochem Pharmacol. (2021) 180:85–117. doi: 10.1007/112_2021_60 34031738

[39] Willems E Gloerich J Suppers A van der Flier M van den Heuvel LP van de Kar N. Impact of infection on proteome-wide glycosylation revealed by distinct signatures for bacterial and viral pathogens. iScience. (2023) 26:107257. doi: 10.1016/j.isci.2023.107257 37520696 PMC10384227

[40] National Cancer Center J. Japanese Guideline for Colorectal Cancer Screening (2005). Available online at: http://canscreen.ncc.go.jp/guideline/daicyougan.html (Accessed April 1, 2025).

[41] Robertson DJ Lee JK Boland CR Dominitz JA Giardiello FM Johnson DA. Recommendations on fecal immunochemical testing to screen for colorectal neoplasia: a consensus statement by the US Multi-Society Task Force on colorectal cancer. Gastrointest Endosc. (2017) 85:2–21.e3. doi: 10.1016/j.gie.2016.09.025 27769516

[42] Thompson EV Snyder JR. Recognition and management of colonic perforation following endoscopy. Clin Colon Rectal Surg. (2019) 32:183–9. doi: 10.1055/s-0038-1677024 PMC6494611 31061648

[43] Kavic SM Basson MD. Complications of endoscopy. Am J Surg. (2001) 181:319–32. doi: 10.1016/S0002-9610(01)00589-X 11438266

[44] Wong HBW Lim GHL. Measures of diagnostic accuracy: sensitivity, specificity, PPV and NPV. Proc Singapore Healthcare. (2011) 20:316–8. doi: 10.1177/201010581102000411

[45] Hisamichi S Fukao A Fujii Y Tsuji I Komatsu S Inawashiro H. Mass screening for colorectal cancer in Japan. Cancer Detect Prev. (1991) 15:351–6. 1751945

[46] Alonso-Garcia V Chaboya C Li Q Le B Congleton TJ Florez J. High mannose N-glycans promote migration of bone-marrow-derived mesenchymal stromal cells. Int J Mol Sci. (2020) 21(19):7194. doi: 10.3390/ijms21197194 33003435 PMC7582662

[47] Yamamoto T Nakayama J Urabe F Ito K Nishida-Aoki N Kitagawa M. Aberrant regulation of serine metabolism drives extracellular vesicle release and cancer progression. Cell Rep. (2024) 43:114517. doi: 10.1016/j.celrep.2024.114517 39024098

[48] Hong CS Funk S Muller L Boyiadzis M Whiteside TL. Isolation of biologically active and morphologically intact exosomes from plasma of patients with cancer. J Extracell Vesicles. (2016) 5:29289. doi: 10.3402/jev.v5.29289 27018366 PMC4808740

[49] Shao H Im H Castro CM Breakefield X Weissleder R Lee H. New technologies for analysis of extracellular vesicles. Chem Rev. (2018) 118:1917–50. doi: 10.1021/acs.chemrev.7b00534 PMC6029891 29384376

[50] Desgagné V Bouchard L Guérin R. microRNAs in lipoprotein and lipid metabolism: from biological function to clinical application. Clin Chem Lab Med. (2017) 55:667–86. doi: 10.1515/cclm-2016-0575 27987357

[51] EVmiRNA: The Extracellular Vesicles miRNA Database . Available online at: https://guolab.wchscu.cn/EVmiRNA/!/ (Accessed July 5, 2025).

[52] Arroyo JD Chevillet JR Kroh EM Ruf IK Pritchard CC Gibson DF. Argonaute2 complexes carry a population of circulating microRNAs independent of vesicles in human plasma. Proc Natl Acad Sci U S A. (2011) 108:5003–8. doi: 10.1073/pnas.1019055108 PMC3064324 21383194

[53] Yi L Zhong X Chen Z Wang Q Yan Y Wang J. MicroRNA-147b promotes proliferation and invasion of human colorectal cancer by targeting RAS oncogene family (RAP2B). Pathobiology. (2019) 86:173–81. doi: 10.1159/000495253 31121595

[54] Carter JV O'Brien SJ Burton JF Oxford BG Stephen V Hallion J. The microRNA-200 family acts as an oncogene in colorectal cancer by inhibiting the tumor suppressor RASSF2. Oncol Lett. (2019) 18:3994–4007. doi: 10.3892/ol.2019.10753 31565080 PMC6759516

[55] Feng YH Tsao CJ. Emerging role of microRNA-21 in cancer. BioMed Rep. (2016) 5:395–402. doi: 10.3892/br.2016.747 27699004 PMC5038362

[56] Cui M Liu Z Wang S Bae S Guo H Zhou J. CRISPR-based dissection of microRNA-23a ~ 27a ~ 24–2 cluster functionality in hepatocellular carcinoma. Oncogene. (2024) 43:2708–21. doi: 10.1038/s41388-024-03115-z, PMID: 39112518 PMC11364504

[57] Li X Wei Y Wang Z. microRNA-21 and hypertension. Hypertens Res. (2018) 41:649–61. doi: 10.1038/s41440-018-0071-z 29973661

[58] Bakhsh T Alhazmi S Farsi A Yusuf AS Alharthi A Qahl SH. Molecular detection of exosomal miRNAs of blood serum for prognosis of colorectal cancer. Sci Rep. (2024) 14:8902. doi: 10.1038/s41598-024-58536-3 38632250 PMC11024162

[59] Matsui Y Togayachi A Sakamoto K Angata K Kadomatsu K Nishihara S. Integrated systems analysis deciphers transcriptome and glycoproteome links in alzheimer's disease. bioRxiv. (2024). doi: 10.1101/2023.12.25.573290

