# Supplementary material for: MicroRNAs in circulating extracellular vesicles as biomarkers of early colorectal cancer captured using high mannose N-glycan-specific lectin from Oscillatoria Agardhii
Source: Front Oncol. 2025 Jul 30;15:1619460. doi: 10.3389/fonc.2025.1619460 (PMC12343284; doi:10.3389/fonc.2025.1619460)
Supplement: Supplementary file 1 [file DataSheet1.docx]

Supplementary Material

# Supplementary Methods

## Comprehensive Profiling of miRNAs

For the comprehensive profiling of miRNAs in OAA1-captured samples, we entrusted Takara Bio Inc. (Japan) with miRNA quantification analysis using the miRCURY platform and miRNome panels (QIAGEN, Germany) covering 752 human miRNAs. Pooled plasma samples from 10 patients with CRC (stage II–IV) or 10 healthy controls were applied to an OAA1-column, and OAA1-captured miRNAs were extracted and purified. The relative expression levels of the target miRNAs were normalized to that of cel-miR-39 using the ΔΔCT method. We compiled a miRNA list with the relative values compared to healthy individuals categorized as follows: 'Fold up' for values above 1.5, 'Stable' for values between 0.7 and 1.3, and 'Fold down' for values below 0.1 (Supplementary Table 2).

## Mixture Experiments

One hundred and fifty microliters of mixed plasma samples from healthy and patients with CRC with mixing ratios (1:0, 2:1, 1:1, 1:2, and 0:1) (see Supplementary Figure 1 for details) were applied to an OAA1-column. Ten miRNA assays were performed for each sample.

## Bioinformatic Analysis

Candidate target genes of the miRNAs were comprehensively identified using the TargetScan database (https://www.targetscan.org/vert_80/). From this list, genes related to the CRC pathway were identified using the KEGG Mapper Search (https://www.genome.jp/kegg/mapper/search.html) (Supplementary Table 6).

# Supplementary Figures and Tables

## Supplementary Figures


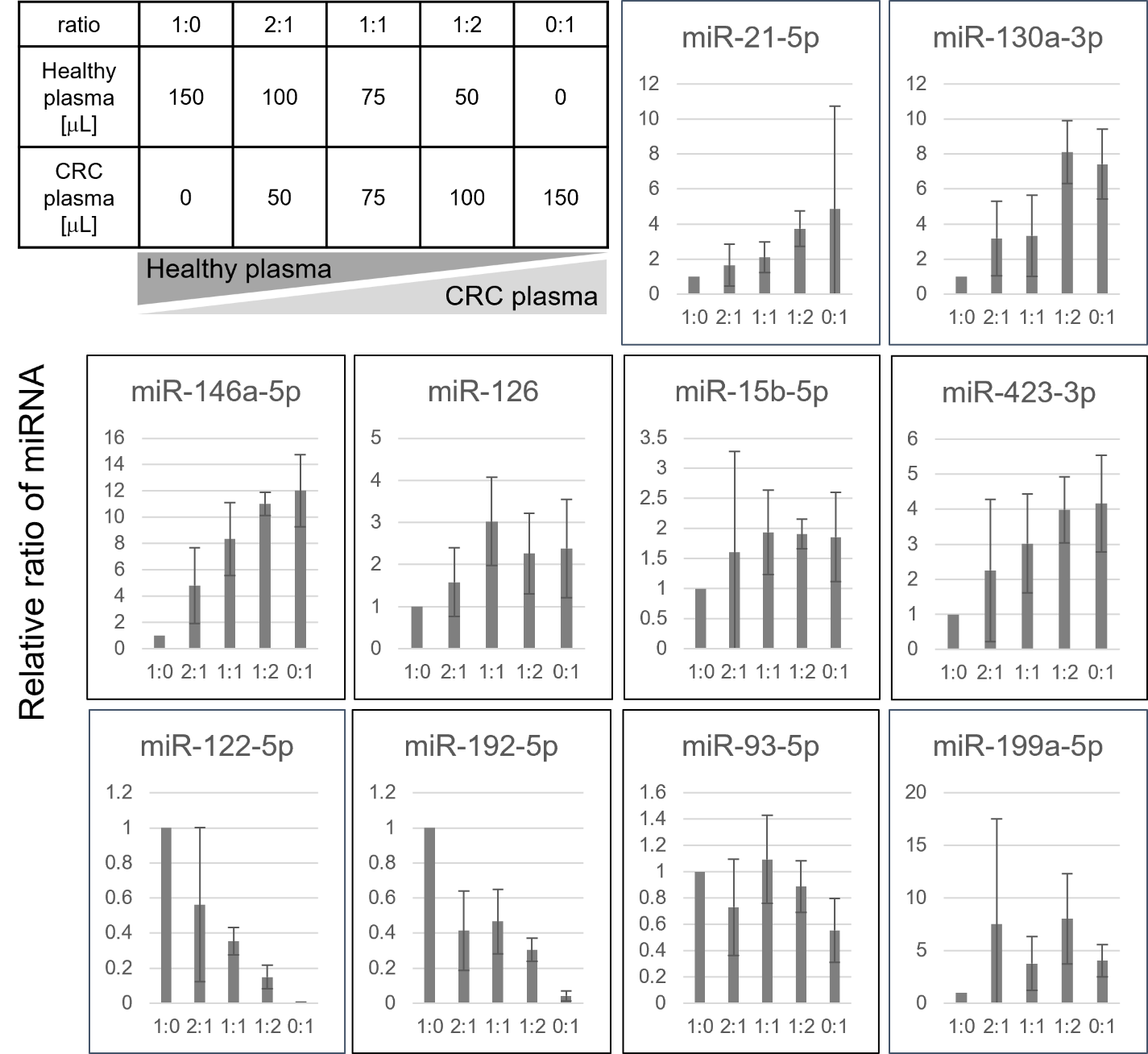


**Supplementary Figure 1.** Mixture experiments of healthy and CRC plasma samples. The pool healthy plasma and the pool CRC plasma were mixed in the prescribed ratio to obtain mixed plasma **(upper left)**. Each miRNA abundance in OAA1-captured EVs from each mixed plasma is shown in relative ratio (healthy plasma:CRC plasma = 1:0). Data represent means ± SD of three independent experiments.


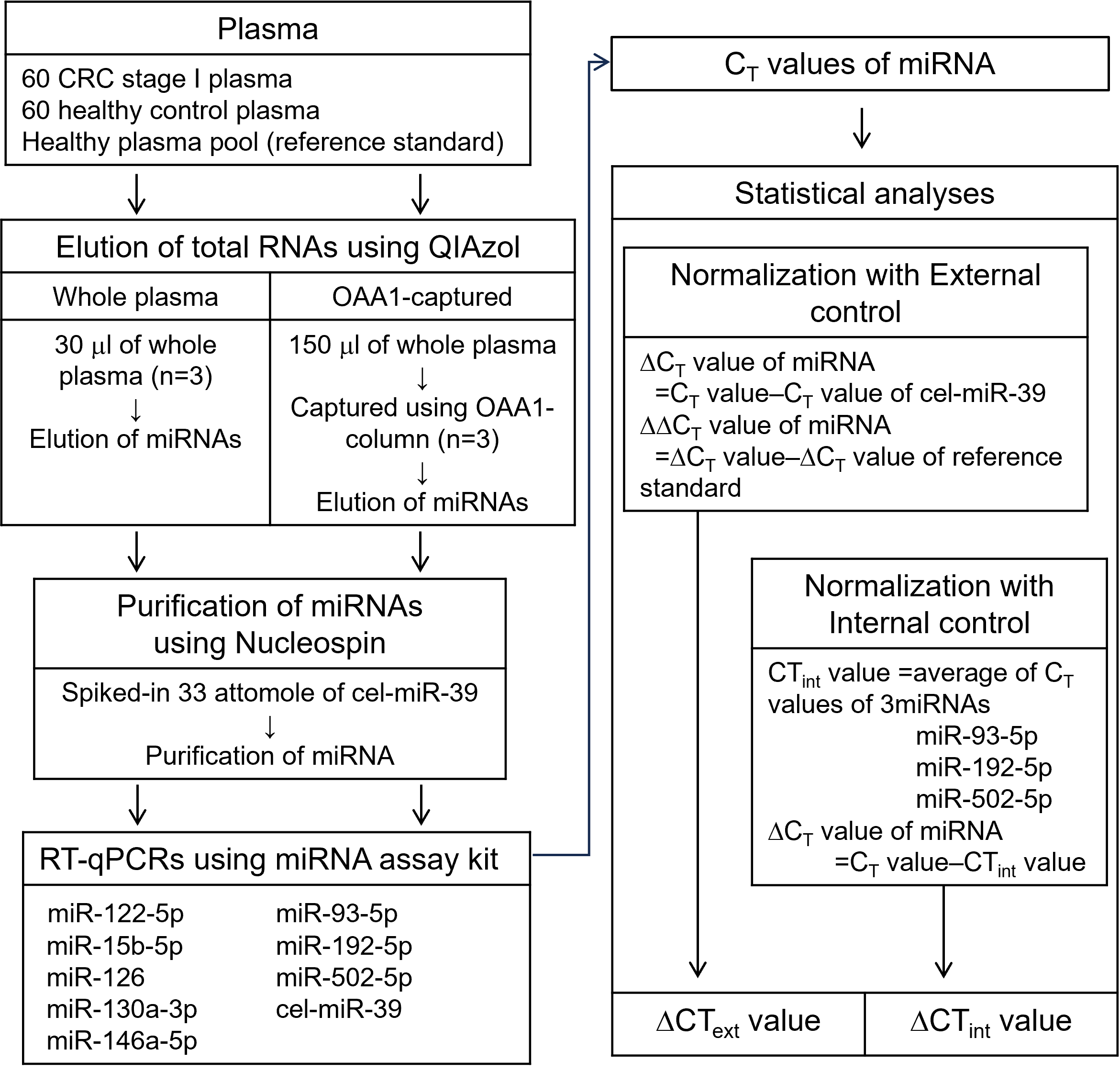


**Supplementary Figure 2.** Experimental flowchart for statistical analysis of data. Sixty healthy control plasma and 60 CRC stage I plasma samples were used, and a commercial healthy plasma pool was used as a standard reference to normalize for variations between experiments. Eight miRNAs and spiked-in cel-miR-39 in the extraction from whole plasma or OAA1-captured EVs were quantified by RT-qPCR, providing C_T_ values for each miRNA. The C_T_ values were normalized using two methods for the following statistical analyses. ΔCT_ext_ and ΔCT_int_ values were normalized using external and internal controls, respectively. (See Materials and Methods for detailed methods)

## Supplementary Tables

**Supplementary Table 1.** TaqMan miRNA assay ID and miRBase accession number of miRNAs

| miRNA | TaqMan miRNA assay ID | miRBase Accession No. |
| --- | --- | --- |
| miR-21-5p | 000397 | MIMAT0000076 |
| miR-130a-3p | 000454 | MIMAT0000425 |
| miR-146a-5p | 000468 | MIMAT0000449 |
| miR-122-5p | 002245 | MIMAT0000421 |
| miR-15b-5p | 000390 | MIMAT0000417 |
| miR-126 | 000450 | MIMAT0000445 |
| miR-423-3p | 002626 | MIMAT0001340 |
| miR-199a-3p | 002304 | MIMAT0000232 |
| miR-93-5p | 001090 | MIMAT0000093 |
| miR-192-5p | 000491 | MIMAT0000222 |
| miR-502-5p | 001109 | MIMAT0002958 |

**Supplementary Table 2.** Profiling of miRNAs in OAA1-captured EVs of CRC plasma compared with those of healthy plasma

| Fold up | |  | Stable | |  | Fold down | |
| --- | --- | --- | --- | --- | --- | --- | --- |
| miRNA | ratio |  | miRNA | ratio |  | miRNA | ratio |
| hsa-miR-221-3p | 170.712 |  | hsa-miR-7-5p | 1.240 |  | hsa-miR-133a-3p | 0.090 |
| hsa-let-7d-3p | 48.349 |  | hsa-let-7a-5p | 1.000 |  | hsa-miR-140-3p | 0.085 |
| hsa-miR-199a-3p | 30.388 |  | hsa-miR-502-5p | 0.940 |  | hsa-miR-451a | 0.067 |
| hsa-miR-9-5p | 8.086 |  | hsa-miR-22-3p | 0.908 |  | hsa-miR-885-5p | 0.063 |
| hsa-miR-28-5p | 7.187 |  | hsa-miR-17-5p | 0.847 |  | mmu-miR-378a-3p | 0.053 |
| hsa-miR-146a-5p | 6.498 |  | hsa-miR-218-1-3p | 0.827 |  | hsa-miR-505-3p | 0.052 |
| hsa-miR-132-3p | 6.433 |  | hsa-miR-1972 | 0.827 |  | hsa-miR-215-5p | 0.037 |
| hsa-miR-21-5p | 5.502 |  | hsa-miR-139-3p | 0.827 |  | hsa-miR-100-5p | 0.025 |
| hsa-miR-15b-5p | 5.063 |  | hsa-miR-1471 | 0.827 |  | hsa-miR-193b-3p | 0.024 |
| hsa-miR-423-3p | 4.563 |  | hsa-miR-222-3p | 0.812 |  | hsa-miR-194-5p | 0.021 |
| hsa-miR-23a-3p | 4.170 |  | hsa-miR-320a | 0.801 |  | hsa-miR-122-5p | 0.019 |
| hsa-miR-877-3p | 4.081 |  | hsa-miR-93-5p | 0.732 |  | hsa-miR-483-5p | 0.017 |
| hsa-miR-195-5p | 3.594 |  |  |  |  | hsa-miR-192-5p | 0.017 |
| hsa-miR-126 | 3.317 |  |  |  |  |  |  |
| hsa-miR-328-3p | 3.294 |  |  |  |  |  |  |
| hsa-miR-24-3p | 3.249 |  |  |  |  |  |  |
| hsa-miR-130a-3p | 1.705 |  |  |  |  |  |  |

**Supplementary Table 3.** Statistical analysis of eight miRNAs in OAA1-captured EVs between 60 healthy controls and 60 patients with stage I CRC

|  | 60 healthy controls | | | | | | | | |
| --- | --- | --- | --- | --- | --- | --- | --- | --- | --- |
|  | ΔCT_ext_ | | | |  | ΔCT_int_ | | | |
| miRNAs | Mean | Med | 25-75% percentile | SD |  | Mean | Med | 25-75% percentile | SD |
| miR-122-5p | 3.24 | 3.39 | [2.03–4.48] | 1.71 |  | -0.74 | -0.84 | [-1.57–0.19] | 1.24 |
| miR-130a-3p | -1.83 | -1.17 | [-3.58–-0.29] | 2.02 |  | 0.27 | 0.25 | [-0.89–1.37] | 1.46 |
| miR-146a-5p | -2.57 | -2.18 | [-4.97–-0.41] | 2.58 |  | -2.15 | -1.81 | [-4.19–-0.39] | 2.11 |
| miR-15b-5p | -2.97 | -2.32 | [-5.47–-0.65] | 2.68 |  | -0.66 | -0.54 | [-2.64–0.89] | 2.26 |
| miR-126 | -2.93 | -2.31 | [-5.66–-0.74] | 2.56 |  | -2.39 | -1.93 | [-4.47–-0.73] | 2.12 |
| miR-93-5p | -0.25 | -0.16 | [-2.00–1.58] | 1.89 |  | -3.19 | -2.93 | [-3.96–-2.47] | 1.03 |
| miR-192-5p | 1.72 | 1.77 | [1.17–2.38] | 1.08 |  | 1.44 | 1.48 | [ 1.08–1.81] | 0.47 |
| miR-502-5p | -0.50 | -0.40 | [-1.26–0.22] | 1.05 |  | 1.75 | 1.64 | [ 1.11–2.38] | 0.89 |

ΔCT_ext_, C_T_ value normalized with external control (corresponding to Figure 4A); ΔCT_int_, C_T_ value normalized with internal control. CRC, colorectal cancer; Med, median (50% percentile); SD, standard deviation. ΔCT_ext_ = Patient ΔC_T_ (C_TmiR-XX_ – C_Tcel-miR-39_) – Reference Standard ΔC_T_ (C_TmiR-XX_ – C_Tcel-miR-39_). ΔCT_int_ = C_TmiR-XX_ – average C_T_ value of miR-93-5p, miR-192-5p and miR-502-5p.

|  | 60 patients with stage I CRC | | | | | | | | |
| --- | --- | --- | --- | --- | --- | --- | --- | --- | --- |
|  | ΔCT_ext_ | | | |  | ΔCT_int_ | | | |
| miRNAs | Mean | Med | 25-75% percentile | SD |  | Mean | Med | 25-75% percentile | SD |
| miR-122-5p | 5.26 | 5.21 | [4.28–6.55] | 1.76 |  | 1.62 | 1.80 | [ 0.68–2.57] | 1.78 |
| miR-130a-3p | -3.88 | -3.82 | [-5.09–-2.74] | 1.67 |  | -1.37 | -1.58 | [-2.07–-0.80] | 1.04 |
| miR-146a-5p | -5.09 | -5.04 | [-6.20–-4.03] | 1.59 |  | -4.28 | -4.45 | [-4.94–-3.66] | 0.90 |
| miR-15b-5p | -5.12 | -5.46 | [-6.33–-4.24] | 1.66 |  | -2.34 | -2.58 | [-3.31–-1.61] | 1.30 |
| miR-126 | -4.99 | -5.25 | [-6.17–-3.95] | 1.59 |  | -4.01 | -4.28 | [-4.89–-3.46] | 1.21 |
| miR-93-5p | -1.74 | -1.87 | [-2.49–-0.97] | 1.22 |  | -4.24 | -4.34 | [-4.92–-3.74] | 0.77 |
| miR-192-5p | 1.64 | 1.71 | [0.96–2.33] | 1.00 |  | 1.77 | 1.80 | [ 1.59–2.03] | 0.40 |
| miR-502-5p | -0.24 | -0.20 | [-0.81–0.27] | 0.70 |  | 2.47 | 2.57 | [ 2.07–3.05] | 0.72 |

**Supplementary Table 4.** Multicollinearity of miRNAs in the logistic regression analysis (variance inflation factor)

|  | Normalized with external control (ΔCT_ext_) | |  | Normalized with internal control (ΔCT_int_) | |
| --- | --- | --- | --- | --- | --- |
|  | OAA1-captured | Whole plasma |  | OAA1-captured | Whole plasma |
| miR-122-5p | 1.334 | 1.244 |  | 1.148 | 1.053 |
| miR-130a-3p | 4.856 | 3.431 |  | 2.324 | 1.959 |
| miR-146a-5p | 9.841 | 5.605 |  | 13.398 | 5.146 |
| miR-15b-5p | 28.629 | 4.354 |  | 23.033 | 2.974 |
| miR-126 | 23.060 | 3.791 |  | 17.580 | 2.501 |

ΔCT_ext_ = Patient ΔC_T_ (C_TmiR-XX_ – C_Tcel-miR-39_) – Reference Standard ΔC_T_ (C_TmiR-XX_ – C_Tcel-miR-39_). ΔCT_int_ = C_TmiR-XX_ – average C_T_ value of miR-93-5p, miR-192-5p and miR-502-5p.

**Supplementary Table 5.** Logistic Regression Analysis for CRC stage I

|  | vif. | Estimate | SE | *z*-value | *p*-value | odds ratio [95% CI] | AIC |
| --- | --- | --- | --- | --- | --- | --- | --- |
| Normalized with external control (ΔCT_ext_) | | | | | | | |
| OAA1-captured | | | | | | | 96.62 |
| (Intercept) |  | -6.3376 | 1.1712 | -5.411 | 6.26E-08 | 0.0018 [0.0002–0.0176] |  |
| miR-122-5p | 1.285 | 0.9138 | 0.1891 | 4.833 | 1.35E-06 | 2.49 [1.72–3.61] |  |
| miR-146a-5p | 1.285 | -0.6658 | 0.1363 | -4.883 | 1.04E-06 | 0.514 [0.393–0.671] |  |
| Whole plasma | | | | | | | 121.14 |
| (Intercept) |  | -3.3897 | 0.7452 | -4.549 | 5.4E-06 | 0.034 [0.078–0.145] |  |
| miR-122-5p | 1.056 | 0.4398 | 0.1428 | 3.08 | 0.00207 | 1.55 [1.17–2.05] |  |
| miR-146a-5p | 3.484 | -1.095 | 0.255 | -4.293 | 1.76E-05 | 0.335 [0.203–0.552] |  |
| miR-15b-5p | 3.409 | 0.5106 | 0.2543 | 2.008 | 0.04468 | 1.67 [1.01–2.74] |  |
| Normalized with internal control (ΔCT_int_) | | | | | | | |
| OAA1-captured | | | | | | | 84.18 |
| (Intercept) |  | -3.6867 | 1.8582 | -1.984 | 0.04725 | 0.025 [0.0007–0.956] |  |
| miR-122-5p | 1.149 | 0.8246 | 0.2112 | 3.904 | 9.45E-05 | 2.28 [1.51–3.45] |  |
| miR-130a-3p | 2.326 | -0.9032 | 0.4258 | -2.121 | 0.03392 | 0.405 [0.176–.934] |  |
| miR-146a-5p | 13.405 | -3.1605 | 0.8405 | -3.76 | 0.00017 | 0.0424 [0.0082–0.22] |  |
| miR-126 | 23.041 | 1.8627 | 0.9563 | 1.948 | 0.05144 | 6.44 [0.988–42] |  |
| miR-15b-5p | 17.579 | 1.0473 | 0.7341 | 1.427 | 0.15366 | 2.85 [0.676–12] |  |
| Whole plasma | | | | | | | 107.24 |
| (Intercept) |  | -1.6678 | 0.6829 | -2.442 | 0.0146 | 0.189 [0.0495–0.719] |  |
| miR-122-5p | 1.014 | 0.2949 | 0.1315 | 2.242 | 0.02496 | 1.34 [1.04–1.74] |  |
| miR-146a-5p | 4.448 | -2.4882 | 0.5537 | -4.494 | 7.00E-06 | 0.0831 [0.0281–0.246] |  |
| miR-126 | 2.925 | 1.0543 | 0.3991 | 2.641 | 0.00826 | 2.87 [1.31–6.28] |  |
| miR-15b-5p | 2.390 | 0.5905 | 0.3243 | 1.821 | 0.06862 | 1.80 [0.956–3.41] |  |

miRNAs were selected by forward-backward stepwise selection method. vif, variance inflation factor; SE, standard error; CI, confidence interval; AIC, Akaike’s information criterion. ΔCT_ext_ = Patient ΔC_T_ (C_TmiR-XX_ – C_Tcel-miR-39_) – Reference Standard ΔC_T_ (C_TmiR-XX_ – C_Tcel-miR-39_). ΔCT_int_ = C_TmiR-XX_ – average C_T_ value of miR-93-5p, miR-192-5p and miR-502-5p.

**Supplementary Table 6.** Target gene candidates in CRC pathway of five miRNAs

|  | Target genes in CRC pathway* | | | | |
| --- | --- | --- | --- | --- | --- |
|  | hsa-miR-122-5p | hsa-miR-126-3p | hsa-miR-15b-5p | hsa-miR-130a-3p | hsa-miR-146a-5p |
| Wnt signaling pathway |  | LRP6 | AXIN2 | MCC |  |
| PI3K-Akt signaling pathway |  |  | KRAS |  | PIK3CB |
| MAPK signaling pathway |  |  | KRAS | DLC1 |  |
| TGF-beta signaling pathway |  |  | SMAD7 | TGFBR2 | SMAD4 |
| ErbB signaling pathway |  |  |  | TGFA, EREG |  |
| mTOR signaling pathway |  | LRP6 | KRAS |  | NRAS |
| p53 signaling pathway | TP5 | BAK1 |  |  |  |
| MSI pathway |  |  | AXIN2, KRAS | TGFBR2 |  |
| Apoptosis |  |  |  |  | APPL1 |
| Cell cycle |  |  | CCND1 |  |  |
| Adherens junction |  |  | PTPRJ | PTPRJ |  |
| Total score of target genes** | 220 | 26 | 1508 | 1029 | 275 |

*KEGG Mapper Search URL: https://www.genome.jp/kegg/mapper/search.html, **Target Scan Human URL: https://www.targetscan.org/vert_71/
